# Bitter Melon Powder Enhances Antioxidant Capacity, Muscle Nutrition, and Glucolipid Metabolic Homeostasis in *Cyprinus carpio* Fed High-Starch Diets

**DOI:** 10.1155/anu/9209833

**Published:** 2025-11-29

**Authors:** Yuru Zhang, Haiying Fan, Yiman Zhang, Kedi Gao, Shibo Zhang, Xianglin Cao, Xinxin Xu, Ronghua Lu

**Affiliations:** College of Fisheries, Henan Normal University, Xinxiang, Henan, China

**Keywords:** antioxidant capacity, bitter melon powder, *Cyprinus carpio*, glucolipid

## Abstract

Carbohydrates in aquaculture feeds can induce metabolic disturbance when exceeding fish utilization capacity, leading to lipid accumulation and insulin resistance. Bitter melon (BM; *Momordica charantia*), rich in saponins, flavonoids, and polysaccharides, shows potential as a functional feed additive for glycemic control and lipid metabolism modulation. This investigation systematically assessed the effects of BM powder (BMP) supplementation (at 0.5%, 1%, and 1.5%) in high-starch (HG) diets for *Cyprinus carpio*. Compared to the HG group, BMP supplementation significantly reduced serum glucose (GLU) and triglycerides (TGs), while elevating total cholesterol (TC) and high-density lipoprotein cholesterol (HDL-C). The BMP group exhibited reduced malondialdehyde (MDA), enhanced antioxidation ability, and mitigated hepatopancreatic and intestinal histopathological damage from carbohydrate overload. Meanwhile, BMP restored muscle C20:3*n*−6 and C22:6*n*−3 (DHA) levels decreased by HG diets. Notably, 1.5% BMP decreased hepatopancreatic and muscular glycogen/lipid deposition, induced by a high-carbohydrate diet. Gene expression analysis revealed BMP upregulated glycolysis-related genes (glucokinase [*gk*], pyruvate kinase [*pk*], and *pfk*) across tissues (hepatopancreas, muscle, intestine, and adipose tissue), while suppressing glycogen synthesis (*gys*) and gluconeogenesis (*g6pase*) genes. Besides, lipid biosynthesis genes were downregulated, corroborating reduced ectopic lipid storage. Taken together, these findings demonstrate that BMP supplementation significantly improves glycemic control, lipid metabolism, and antioxidant capacity in common carp. This suggests that BMP could serve as a natural, sustainable aquafeed additive to counter metabolic syndrome in intensively farmed fish.

## 1. Introduction

Carbohydrates, also known as sugars, are essential energy sources in fish feed. Studies have shown that an appropriate level of carbohydrates can promote fish growth, improve intestinal health, and enhance immune function. For instance, a higher carbohydrate content in feed has been shown to promote the growth of *Micropterus salmoides* [1] and increase the healthy bacteria in Chinese Perch [2]. However, fish have a limited ability to metabolize sugars. Excessive carbohydrate intake delays the return of blood glucose (GLU) levels to normal, causing prolonged hyperglycemia and promoting fat storage, both of which contribute to metabolic disorders [3]. These conditions reduce disease resistance [4], impair growth [5], and increase mortality in fish, thereby significantly affecting the economic viability of the aquaculture industry [6–8]. Consequently, researchers have concentrated on developing and implementing scientific and effective strategies to improve fish carbohydrate and lipid metabolism.

*Momordica charantia* L., commonly known as bitter melon (BM), is a multifunctional plant, rich in nutrients including carbohydrates, proteins, and lipids [9]. It also contains a variety of bioactive compounds, such as flavonoids, alkaloids, triterpenoids, saponins, sterols, and others. BM has long been utilized as both a medicinal plant and a vegetable [10]. BM and its active compounds exhibit significant hypoglycemic effects [11–14]. For instance, glycemic concentrations in type II diabetes patients were significantly reduced following treatment with BM extract [15] or the BM peptide (mcIRBP-19) [16]. BM or BM extract alleviates diabetes symptoms by regulating disaccharidase activity in the intestines and kidneys of rats [17], reducing pro-inflammatory factors, and preventing insulin resistance through the NF-κB, JNK [18], and AMPK/PI3K pathway [19]. Moreover, BM has demonstrated lipid-lowering effects. Supplementation with BM reduces serum GLU, triglyceride (TG), low-density lipoprotein cholesterol (LDL-C), as well as liver TG and total cholesterol (TC) levels in rats [20–22], while also elevating serum high-density lipoprotein cholesterol (HDL-C) concentrations [23]. After 8 weeks of BM powder (BMP) treatment, high-fat diet rats exhibited inhibited weight gain, improved GLU intolerance, and decreased inflammation in the colon mucosa, liver tissue, and systemic inflammation [24]. Additionally, injecting obese mice with 10 g/kg of BM seed oil significantly reduced body weight and fat content while normalizing serum free fatty acid (FA) levels. At higher doses, serum leptin levels were also normalized [25].

Consistent with findings in mammalian studies, research on fish has demonstrated that BM and its active compounds can effectively reduce blood sugar and lipid levels, inhibit gluconeogenesis, alleviate insulin resistance, and enhance protein metabolism efficiency in common carp (*Cyprinus carpio*) [26]. Specifically, supplementing a high-carbohydrate diet with 1600 mg/kg of BM *charantia* saponins improves protein deposition in carp, mitigates hyperlipidemia symptoms, inhibits gluconeogenesis, and reduces insulin resistance by activating the IRS-1/PI3K/AKT signaling pathway in the hepatopancreas [26]. Additionally, adding BM to the feed of soft-shelled turtles improves feed palatability, offers detoxifying benefits, and meets their nutritional requirements [27]. Therefore, developing BM as an additive in aquatic feed holds significant potential. However, the application of BM in aquatic feed and its impact on glycolipid metabolism are still in the preliminary stages, requiring further in-depth research.

The common carp (*C. carpio*) is a widely distributed freshwater omnivorous species of considerable economic importance, especially in China. According to the most recent FAO [28] statistics, global common carp production reached 31.79 million tons in 2022, representing 51.6% of total world aquaculture production. In recent years, GLU and lipid metabolism disorders associated with feed nutrition have become increasingly prevalent in intensive farming systems. These issues have had a detrimental impact on both the quality of carp farming and the overall development of the aquaculture industry. Therefore, identifying feed additives that can regulate glucolipid metabolism in carp is of both theoretical and practical importance. To investigate the effects of BM on the glycolipid metabolism in common carp, this study investigates the impact of incorporating BMP into high-starch feed on growth performance, nutritional composition, physiological and biochemical markers, FA composition, and key genes involved in carbohydrate and lipid metabolism. The findings could offer valuable insights into the development of BM-based feed additives and deepen our understanding of the mechanisms through which BM regulates glucolipid metabolism in fish.

## 2. Materials and Methods

### 2.1. Experimental Animals and Feed

The common carp were obtained from Lao Wang Fish Farm in Zhengzhou, Henan, and temporarily kept at the aquaculture base of Henan Normal University for 1 month, where they were fed commercial feed. Subsequently, 450 healthy common carp (3.06 ± 0.19 g) were selected for the formal culture experiment. The fish were randomly assigned to five groups, with three tanks/group and 30 fish/tank. During the culture period (8 weeks), the fish were fed three times daily (8:00, 12:00, and 17:00). Water temperature was maintained at 25 ± 3°C and pH levels ranged from 7.3 to 7.8.

Five isonitrogenous and isocaloric feeds were prepared (Table 1), with the control feed containing approximately 27.83% nonnitrogen extract, and the high-starch feed containing approximately 40% nonnitrogen extract. Additionally, 0.5%, 1%, and 1.5% BMP were added to the high-starch feed. BM fruits were purchased from the Xinxiang City Market, sliced (seeds included), freeze-dried, and ground into a powder. All feed ingredients were pulverized using a grinder (Nanjing Feiyu Drying Equipment Co., Ltd, China), sieved through a 60-mesh screen, and mixed uniformly in stages. Water and oil were then added to ensure even distribution. After the feed was prepared, it was air-dried in a cool place and stored at −20°C.

### 2.2. Sample Collection and Processing

All fish-related procedures adhered to the animal ethics requirements of the Institutional Animal Care and the Use Committee at Henan Normal University (approval no. HNSD-2024BS-0308).

After the culture experiment, all fish were fasted for 24 h. Subsequently, sampling was performed on fish anesthetized with 100 mg/LMS-222 (Aladdin, China). Following the methodology outlined in our prior research [29], the body weight and length of the carp were measured. Additionally, the viscera and hepatopancreas (nine fish/tank) were weighed to calculate growth performance indicators. The whole fish and muscle tissues (three fish/tank) were frozen quickly by liquid nitrogen and stored in refrigerator (−20°C) for the analysis of routine nutrients. The blood samples (six fish/tank) were obtained from the tail vein for biochemical analysis. The hepatopancreas, muscle, intestine, and abdominal fat tissues (three fish/tank) were stored in refrigerator (−80°C) for subsequent gene expression analysis.

### 2.3. Histological Analysis of the Hepatopancreas, Intestine, and Muscle

Hepatopancreas, intestine, and muscle samples from three carps per barrel were randomly selected and placed in a 4% paraformaldehyde solution for morphological observation and the assessment of fat and glycogen accumulation [30]. Tissue sections were stained using the glycogen periodic acid-Schiff (PAS) staining kit and hematoxylin and eosin (H&E) staining kit (Solarbio Biotechnology Co., Ltd). Histological images were captured using an Axio Vert A1 inverted microscope manufactured by Carl Zeiss (Germany).

### 2.4. Biochemical Parameter, Conventional Nutrient Composition, and FAs Composition Determination

The contents of GLU, TG, HDL-C, malondialdehyde (MDA), and other biochemical markers were determined following the manufacturer's instructions for the respective kits. These biochemical kits were sourced from Nanjing Jiancheng Bioengineering Institute, China.

Consistent with our prior research [29], the determination of crude fat, crude protein, ash, and moisture was conducted utilizing methods such as the Kjeldahl nitrogen, Soxhlet extraction, dry ash, and dry weight methods, respectively. The identification of FAs was performed using a gas chromatograph (GC) model 7890B (Agilent), which was fitted with a DB-WAX column (dimensions: 15 m × 250 µm × 0.25 µm, Agilent) [31]. A standard mixture of 37 FA methyl esters (Supelco, Merck KGaA, Darmstadt, Germany) was utilized for the identification of FA composition, and the relative percentage of each FA was calculated through area normalization.

### 2.5. RT-qPCR Assays

RNA was extracted according to the kit instructions of RNAiso Plus. The assessment of RNA purity and concentration was conducted by evaluating the OD260/280 ratio utilizing a microvolume nucleic acid and protein detector. cDNA was then synthesized following the protocols outlined in operating manual of PrimeScript RT Reagent Kit. The genes' cDNA sequences were downloaded from the NCBI database, and specific gene primers for RT-PCR (Table 2) were generated by Primer5.0 software and produced by Sangon Biotech Co., Ltd (China). RT-PCR was conducted following the instructions for the 2×SYBR qPCR Master Mix. The relative expression levels of each gene were determined by the 2^−*ΔΔ*Ct^ method [32].

### 2.6. Data Analysis and Processing

Experimental data are presented as the mean ± standard error of the mean (SEM). One-way analysis of variance (ANOVA) followed by Duncan's multiple range test for multiple comparisons was conducted by SPSS version 26.0. Statistical figures were generated by GraphPad Prism (version 8.0). The heatmap depicting the Pearson correlation between different indices was generated using an online platform for visualization (https://www.bioinformatics.com.cn).

## 3. Results

### 3.1. The Effect of BMP on the Growth Indicators of Carp

No statistically significant differences were detected in the final body weight (FBW; 10.12–11.43 g), weight gain rate (WGR; 231.10%–273.94%), specific growth rate (SGR; 2.14%–2.35%), or feed conversion ratio (FCR; 1.60–1.87) of carp across the five groups (*p* > 0.05). Following the high-starch diet, the hepatosomatic index (HSI) of carp significantly increased to 2.45% (*p* < 0.05), but returned to control levels upon BMP supplementation. Notably, the 0.5% and 1.5% BMP groups exhibited a statistically significant reduction in comparison to the HG group (*p* < 0.05; Table 3).

### 3.2. The Impact of BMP on Biochemical Indicators in the Serum and Hepatopancreas of Carp

**Figure 1:**
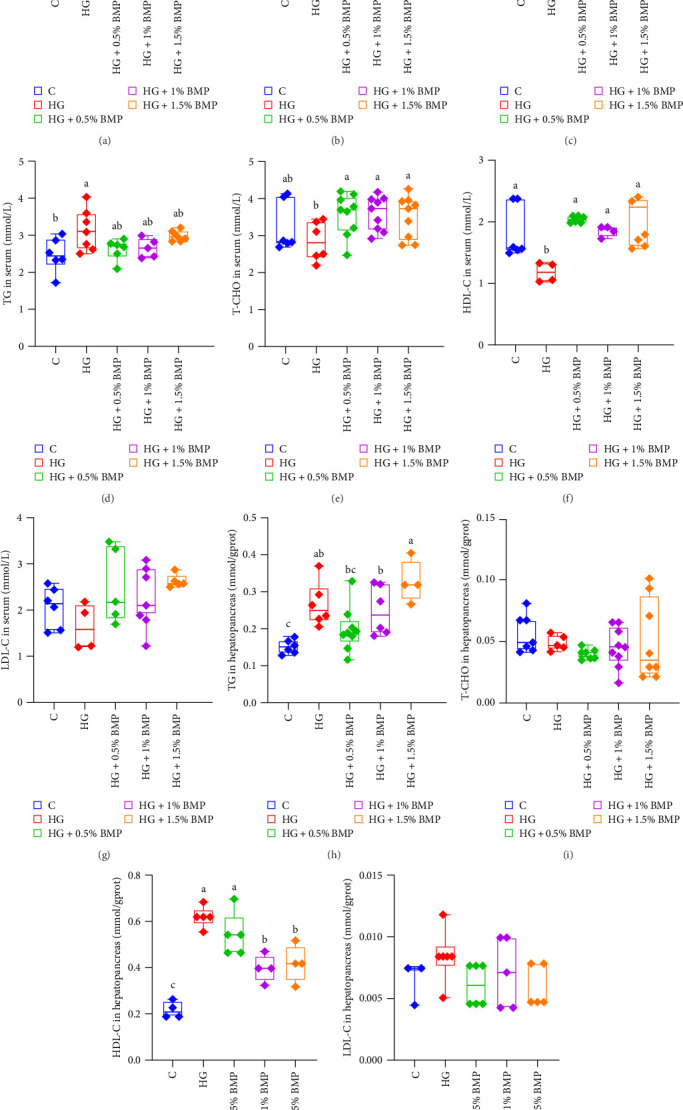
The impact of BMP on biochemical indices in common carp. Subparts (A–G) represent serum indices, while Subparts (H–K) represent hepatopancreas indices. The data are expressed as the mean ± standard error of the mean (SEM), with a sample size of *n* = 9 for each group. Values that are marked with different superscript letters indicate statistically significant differences (*p* < 0.05).

Biochemical indices in carp serum were analyzed and the results showed that after feeding a high-starch diet, serum GLU content significantly increased to 11.84 nmol/mL; but following the addition of varying proportions of BMP, the blood GLU levels in the carp significantly decreased (*p* < 0.05). The 0.5% and 1% BMP groups showed reductions to 8.04 and 9.36 nmol/mL, respectively, with no significant difference compared to the control group's level (7.81 nmol/mL; Figure 1A). Similarly, the serum TG content in the control group was 2.47 mmol/mL, which statistically increased to 3.14 mmol/mL in the HG group (*p* < 0.05), then, it decreased back to the control level in all BMP addition groups (Figure 1D). However, the serum T-CHO and HDL-C levels followed a different trend, showing a marked decrease in the HG group, followed by a significant increase in the BMP supplementation groups (*p* < 0.05; Figure 1E,F). Following the high-starch diet, the AST content in the serum of carp showed a slight increase (*p* > 0.05); but it declined after BMP addition, with the 1% BMP group exhibiting the lowest value (*p* < 0.05; Figure 1C). Further analysis of biochemical indexes in the hepatopancreas revealed that the TG and HDL-C levels in the carp from the HG group were markedly elevated (*p* < 0.05). While BMP supplementation did not result in a significant reduction in TG levels, a marked decrease in HDL-C levels was observed in the groups receiving 1% and 1.5% BMP (Figure 1H,J).

**Figure 2:**
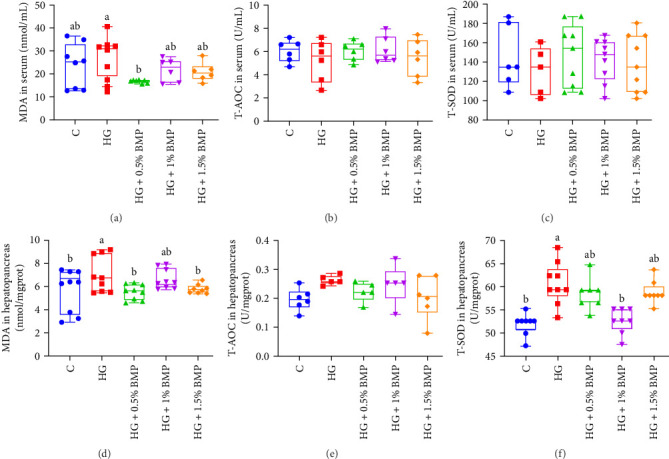
The influence of BMP on the activity of antioxidant enzymes. Subparts (A–C) represent serum enzyme activity indices, while Subparts (D–F) represent hepatopancreas enzyme activity indices. The sample size for each group is *n* ≥ 9. The data and significant differences are consistent with those presented in Figure 1.

The analysis of antioxidant enzyme activities in carp serum indicated that serum MDA concentration slightly increased to 27.82 nmol/mL subjected to high-starch diet (*p* > 0.05); after BMP supplementation, it returned to that observed in the control group, exhibiting a statistically significant reduction in MDA levels (17.22 nmol/mL; *p* < 0.05; Figure 2A). Similarly, when compared to the control group, the concentrations of MDA and T-SOD in the hepatopancreas of the HG group were 7.04 nmol/ML and 60.73 U/mg prot, respectively, representing a statistically significant elevation (*p* < 0.05). However, BMP supplementation reduced both MDA and T-SOD levels effectively (Figure 2D,F).

### 3.3. The Effect of BMP on the Conventional Nutritional Components and FAs Composition of Carp

The content of crude protein and ash content in both the whole fish and muscle did not exhibit statistically significant variations across five groups. The high-starch diet resulted in an increase in the ash content of the muscle; however, this increase reverted to baseline levels following the supplementation of BMP (*p* > 0.05; Table 4).

An examination of the impact of BMP on the FA composition of carp muscle indicated that following a high-GLU diet, the levels of oleic acid (C18:1; 31.36%) in the muscle tissue of carp were markedly elevated in comparison to the control group. After 1.5% BMP supplementation, the concentration of this monounsaturated FA returned to control levels (27.62%; *p* < 0.05). In contrast, the concentrations of linoleic acid (C18:2*n*−6; 18.14%), α-linolenic acid (C18:3*n*−3; 2.08%), eicosatrienoic acid (C20:3*n*−6; 1.51%), eicosatetraenoic acid (C20:3*n*−3; 0.08%), docosahexaenoic acid (C22:6*n*−3; 3.33%), ∑PUFA (26.93%), ∑PUFA/∑SA (0.78%), and ∑*ω*−6 (20.15%) in the HG group were markedly lower than those observed in the control group (*p* < 0.05). Nevertheless, the introduction of 1.5% BMP resulted in a statistical increase in the levels of C20:3*n*−6 (1.67%), C20:3*n*−3 (0.09%), and C22:6*n*−3 (3.52%), restoring them to the contents observed in the control group (*p* < 0.05; Table 5).

Additionally, we also measure the FA content in the hepatopancreas (Supporting Information: Table S1) and intestine (Supporting Information: Table S2). Similar to the changes in unsaturated FAs within muscle tissue, the contents of C20:3*n*−6 (0.81%), C20:3*n*−3 (0.05%), C22:6*n*−3 (1.88%), and ∑*ω*−3 (4.36%) in the intestine samples of the HG group were markedly reduced in comparison to the control cohort. After BMP supplementation, the levels of C20:3*n*−6, C20:3*n*−3, and C22:6*n*−3 increased, but the changes were not statistically significant. After adding 1% BMP, the ∑*ω*−3 content (5.17%) returned to control levels (Supporting Information: Table S2). There were no consistent changes in the FAs composition of the hepatopancreas.

### 3.4. The Effect of BMP on the Deposition of Glycogen and Lipid in the Muscle and Hepatopancreas and on the Intestinal Morphology of Carp

**Figure 3:**
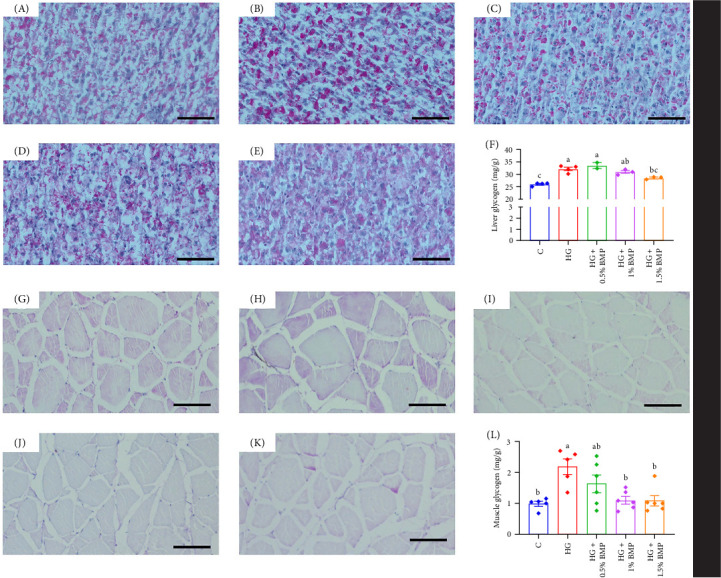
Changes in glycogen content in the hepatopancreas (A–F) and muscle (G–L) of common carp. (A) Control; (B) HG, (C) HG + 0.5% BMP; (D) HG + 1% BMP; (E) HG + 1.5% BMP; (F) hepatopancreas and (L) muscle: glycogen content quantified utilizing a assay kit. (G) Control, (H) HG, (I) HG + 0.5% BMP; (J) HG + 1% BMP, (K) HG + 1.5% BMP, (L) glycogen content measured using a glycogen assay kit. The sample size for each group is *n* ≥ 3. Scale bar: 50 µm.

The findings from histologic section indicated that glycogen content in both the hepatopancreas and muscle significantly increased following a high-starch diet (Figure 3B,H). However, the glycogen content markedly decreased after supplementation with varying proportions of BMP. The results obtained from the glycogen assay kit indicated a significant increase in hepatic (32.16 mg/g) and muscle (2.44 mg/g) glycogen levels in the high-starch group. Furthermore, the supplementation of 1.5% BMP effectively restored glycogen levels to those observed in the control group, with statistical significance (*p* < 0.05; Figure 3F,L).

**Figure 4:**
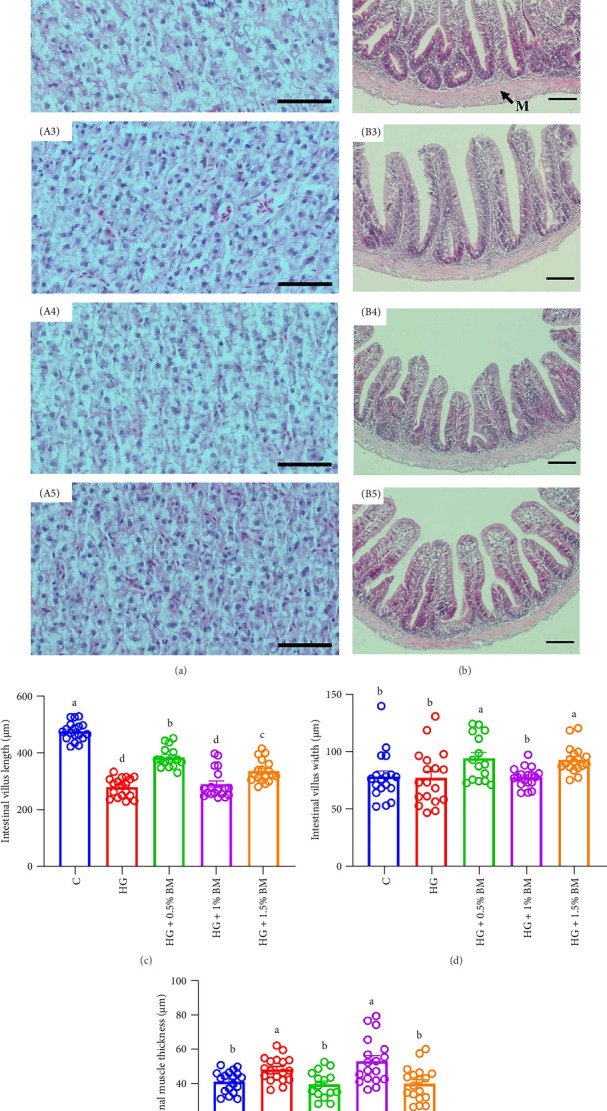
Morphological changes in the hepatopancreas and intestine of common carp. (A) Hepatopancreas: (A1) control, (A2) HG, (A3) HG + 0.5% BMP, (A4) HG + 1% BMP, and (A5) HG + 1.5% BMP. (B) Intestine: (B1) control, (B2) HG, (B3) HG + 0.5% BMP, (B4) HG + 1% BMP, (B5) HG + 1.5% BMP). (C and D) A quantitative data show as the mean ± SEM (*n* ≥ 15 per group) for intestinal villus height (C), villus width (D), and muscle thickness (E). M: muscularis, V: intestinal villus. Scale bar: 50 µm.

The findings of histomorphology demonstrated that hepatocytes of the carp from high-starch group displayed noticeable vacuolation, with nuclei displaced from the cell center and lipid accumulation. After supplementing with different proportions of BMP, lipid accumulation decreased, vacuolation was reduced, and the nuclei gradually returned to a central position (Figure 4A1–A5). In the high-starch group, a notable reduction in the length of intestinal villi was observed (Figure 4B2,C). Conversely, the incorporation of 0.5% and 1.5% BMP resulted in a significant increase in villi length (*p* < 0.05). After high-starch feeding, the thickness of the intestinal muscular layer significantly increased but it decreased significantly and returned to control levels after the addition of 0.5% and 1.5% BMP (*p* < 0.05; Figure 4B–D).

### 3.5. The Influence of BMP on the messenger RNA (mRNA) Expression of Essential Genes Associated With Glycolipid Metabolism in Carp

**Figure 5:**
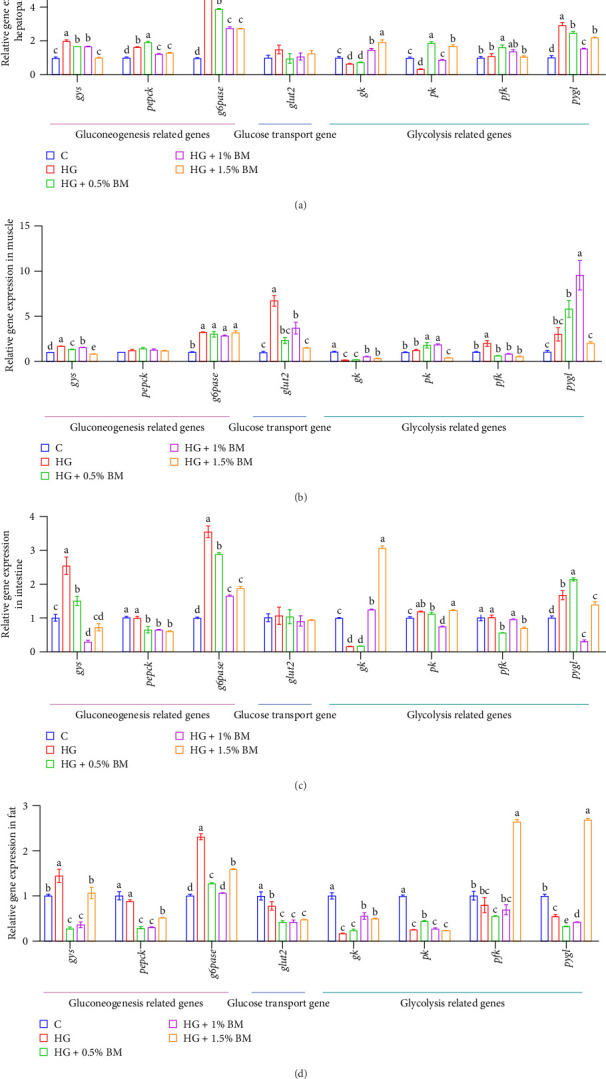
The effects of BMP on the transcription of essential genes participating in glycogen synthesis, glycogenolysis, and transport. (A) Hepatopancreas; (B) muscle; (C) intestine; (D) abdominal fat. The sample size for each tank is 3. The data and significant differences are consistent with those presented in Figure 1.

Following high-starch feeding, the expression levels of essential gluconeogenesis genes (*gys*, *pepck*, and *g6pase*) were significantly elevated in the hepatopancreas. Supplementation with various concentrations of BMP resulted in a notable decrease in the mRNA levels of these genes, with the 1.5% BMP group showing levels similar to those of the baseline group (*p* < 0.05; Figure 5A). Similarly, the mRNA levels of *gys* and *g6pase* in muscle, intestine, and fat tissues were initially upregulated in the HG group but downregulated in the BMP-supplemented groups (Figure 5). Moreover, BMP supplementation at different doses statistically reduced the expression levels of GLU transporter 2 (*glut2*) in muscle and fat tissues (*p* < 0.05; Figure 5B,D). An examination of glycolytic gene expression in different tissues indicated that high-starch feeding resulted in a notable decrease in the expression levels of glucokinase (*gk*) and pyruvate kinase (*pk*) within the hepatopancreas (Figure 5A), with a similar trend observed for *gk* in muscle, intestine, and abdominal adipose tissues (Figure 5C,D). BMP supplementation significantly increased the expression of these genes (*p* < 0.05). Meanwhile, high-starch feeding downregulated the expression of *pk* and glycogen phosphorylase (*pygl*) in fat tissues. However, *pk* expression significantly increased with 0.5% BMP supplementation, while *pygl* expression significantly increased with 1.5% BMP supplementation (*p* < 0.05; Figure 5D). In contrast, a high-starch diet resulted in a notable elevation of *pygl* expression in the hepatopancreas, muscle, and intestine. This expression was significantly reduced following the supplementation of 1.5% BMP (Figure 5).

**Figure 6:**
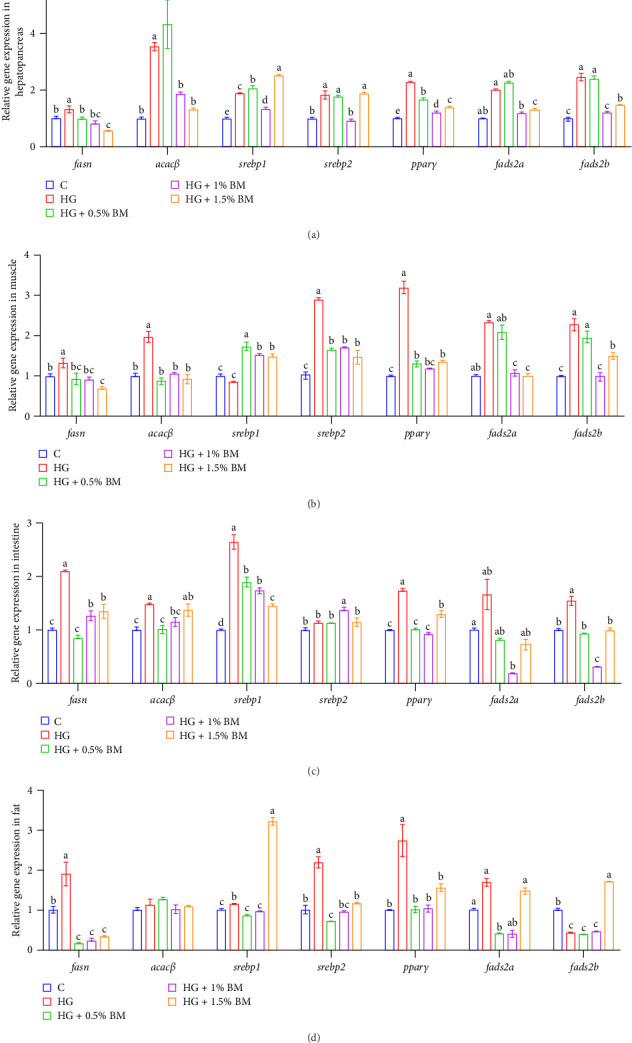
The effects of BMP on the transcription of essential genes participating in in lipid synthesis. The sample size for each tank is 3. The data and significant differences are consistent with those presented in Figure 1. (A) Hepatopancreas; (B) muscle; (C) intestine; (D) abdominal fat.

**Figure 7:**
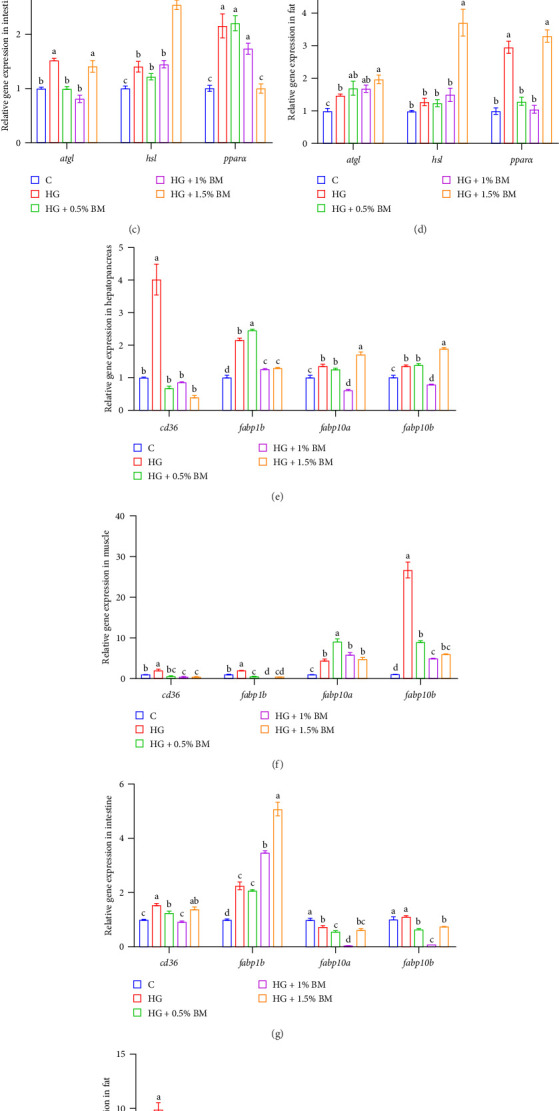
The effects of BMP on the transcription of essential genes participating in lipid degradation (A–D) and transport (E–H). The sample size for each tank is 3. The data and significant differences are consistent with those presented in Figure 1.

In relation to genes associated with lipid synthesis, high-starch feeding markedly elevated the mRNA levels of *fasn*, *acacβ*, and *pparγ* in all four tissues (hepatopancreas, muscle, intestine, and adipose tissue). Conversely, supplementation with 1% and 1.5% BMP markedly downregulated these genes' expression (*p* < 0.05; Figure 6). The mRNA levels of sterol regulatory element-binding protein 2 (*srebp-2*) in muscle and adipose tissues were elevated in the HG group, but these levels decreased significantly after supplementation with all three BMP concentrations (*p* < 0.05; Figure 6B,D). High-starch feeding also significantly increased the transcriptional levels of FA desaturases (*fads2a* and *fads2b*) within the hepatopancreas, muscle, and intestine, and these levels were significantly reduced with 1% and 1.5% BMP supplementation (*p* < 0.05; Figure 6A,B,D). Compared to control group, the levels of expression for lipid catabolism-related genes (*atgl*, *hsl*, and *pparα*) were increased in both the hepatopancreas and intestine of the HG group. Supplementation with 0.5% and 1% BMP significantly decreased their expression in the hepatopancreas (*p* < 0.05). In the intestine, the abundance of *atgl* returned to baseline levels following the addition of 0.5% and 1% BMP, while the mRNA level of *pparα* significantly decreased in the presence of 1.5% BMP (*p* < 0.05; Figure 7C). Lipid catabolic gene analysis in muscle revealed that high-starch feeding inhibited *atgl* expression, but supplementation with 0.5% BMP significantly restored its expression (*p* < 0.05; Figure 7B). In abdominal adipose tissue, a high-starch diet significantly elevated the mRNA abundance of *atgl* and *pparα* (*p* < 0.05); after adding 0.5% and 1% BMP, *pparα* expression decreased statistically, while *atgl* abundance was notably upregulated in the 1.5% BMP group when compared to the high-starch group (*p* < 0.05; Figure 7D). Regarding the mRNA levels of FA transport genes, high-starch feeding significantly increased *cd36* expression across four tissues, from the hepatopancreas to abdominal adipose (Figure 7E,F). Supplementation with BMP at various doses significantly reduced *cd36* abundance varying extents (*p* < 0.05). Under high-starch conditions, *fabp1b* expression was significantly higher in four tissues compared to the control cohort (*p* < 0.05). Supplementation with 1% BMP notably downregulated *fabp1b* expression in the hepatopancreas, muscle, and fat tissues, while its expression in the intestine showed a gradual increase with BMP supplementation at different concentrations. Additionally, the mRNA levels of *fabp10a* and *fabp10b* in muscle tissue were markedly higher in the HG group compared to the baseline group, with BMP supplementation gradually reducing *fabp10b* abundance (*p* < 0.05; Figure 7F).

### 3.6. Integrated Analysis of Physiological and Biochemical Indicators With Gene Expression in the Hepatopancreas

**Figure 8:**
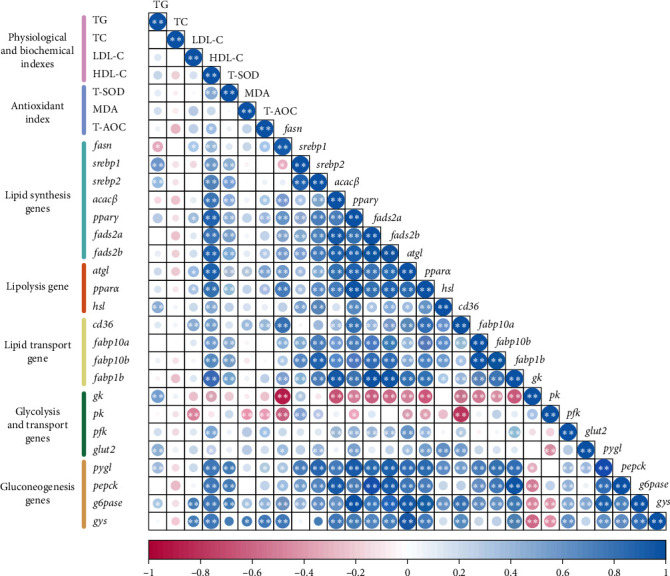
Correlation analysis of hepatopancreas lipid deposition, antioxidant capacity, and the transcription of genes participating in glucose and lipid metabolism. Color depth signifies the magnitude of the correlation, with blue indicating a positive correlation and red indicating a negative correlation. The size of the circle is proportional to the *r* value. An asterisk (*⁣*^*∗*^) denotes a significance level of *p* < 0.05, while two asterisks (*⁣*^*∗∗*^) indicate a significance level of *p* < 0.01.

Correlation analysis reveals a strong association between hepatic lipid deposition, antioxidant capacity, and the mRNA levels of genes associated with the metabolism of GLU and lipid (Figure 8).

TG levels are positively correlated with the transcriptional levels of genes involved in lipolysis (*hsl*), *glycolysis* (*gk*), and *glut2* (*r* > 0.44, *p* < 0.01). Both LDL-C and HDL-C show positive correlations with antioxidant capacity and lipid metabolism-related genes. Notably, HDL-C exhibits a significantly positive correlation with the mRNA expression of lipid synthesis gene (*acacβ*, *pparγ*, *fads2a*, and *fads2b*), lipid degradation genes (*atgl* and pparγ), and lipid transport genes (*fabp1b*; *r* > 0.8, *p* < 0.01). Additionally, LDL-C and HDL-C show an inverse relationship with genes associated with glycolysis (*gk* and *pk*; *r* < −0.25, *p* < 0.01) while showing a positive interaction with genes implicated in gluconeogenesis and GLU transport.

Antioxidant indicators, including T-AOC, MDA, and T-SOD, exhibit positively correlated with the mRNA abundance of genes that regulate lipid metabolism. T-SOD, in particular, shows a strong positive correlation (*p* < 0.01) with lipid synthesis genes (*srebp1*, *srebp2*, *acacβ*, *pparγ*, *fads2a, and fads2b*) and lipid transport genes (*fabp10a*, *fabp10b, and fabp1b*; *r* > 0.45, *p* < 0.01). Similarly, MDA is positively correlated (*r* = 0.31, *p* < 0.05) with the expression of lipid degradation gene (*atgl*). Additionally, T-AOC is negatively correlated with the expression patterns of genes regulating glycolysis (*gk and pk*), while positively associated with the mRNA expression of gluconeogenesis-related genes, particularly *pygl* and *g6pase* (*r* > 0.31, *p* < 0.05).

The mRNA levels of essential genes that regulate GLU and lipid metabolism demonstrate varying degrees of correlation. Except for *srebp1*, the expression of genes tied to lipid synthesis, including *srebp2*, *acacβ*, *pparγ*, *fads2a*, and *fads2b*, is positively correlated with genes associated with lipid degradation and transport (*r* > 0.6, *p* < 0.01). Correlation analysis with GLU metabolism genes indicates that *fasn*, *acacβ*, *pparγ*, *fads2a*, *fads2b*, *atgl*, *pparα*, and *fabp1b* are negatively correlated with *gk* expression but positively correlated with genes involved in gluconeogenesis (*r* < −0.5, *p* < 0.01).

## 4. Discussions

### 4.1. The Addition of BMP Had Minimal Impact on the Overall Growth Characteristics of Carp, but It Reduced the Elevated HSI Caused by the High-Starch Diet

Previous studies have shown that when 0.16% BM saponins are added to high-sugar feed, the WGR and SGR of carp significantly increase [33]. Nevertheless, alternative research has suggested that the growth performance of carp does not show significant differences compared to the control group when different proportions of BM extract are added to the feed [34]. In this trial, the final weight and WGR of carp in the HG group were observed to be lower than those in the control cohort. However, following the supplementation of 0.5% and 1% BMP, there was a slight increase in these parameters. Nonetheless, the differences between the groups were not statistically significant (Table 3), suggesting that BMP has a minimal impact on the performance in growth.

The HSI can be employed to evaluate the health condition of fish, and an increased HSI indicates the occurrence of pathological changes in the hepatopancreas. Research indicates that the consumption of high-energy feed is associated with a rising trend in the HSI of carp [5]. In this study, high-starch diet elevated the HSI, suggesting that high-carbohydrate feed may cause adverse effects such as fat accumulation or swelling in the hepatopancreas of carp. The incorporation of 1.5% BMP resulted in a reduction in both the VSI and HSI of carp when compared to the HG group (Table 3). This suggests that the addition of BMP mitigates, to a certain degree, the negative impacts of high-starch diets on hepatopancreas fat accumulation and hepatomegaly in carp.

### 4.2. The BMP Can Reduce Hyperglycemia and Hyperlipidemia in Carp Induced by a High-Starch Diet, While Simultaneously Enhancing Their Antioxidant Capacity

Studies on mammals have demonstrated that both compound preparations of BM and BM extracts can lower the concentrations of GLU and lipid in the blood of type II diabetic mice and rats [35, 36]. Similarly, the addition of BM saponins significantly lowers GLU and TG levels in juvenile carp [33]. In this study, following high-starch feeding, GLU and TG levels in the carp serum significantly increased, while HDL-C content significantly decreased. However, after the addition of BMP (Figure 1), these parameters returned to the levels observed in the control group, suggesting that BMP effectively alleviates hyperglycemia and hyperlipidemia in carp.

ALT and AST are key indicators of liver inflammation. Elevated levels of these enzymes in the serum and hepatopancreas suggest potential liver cell damage. Studies in both fish and mammals have demonstrated that active components of BM, such as BM saponins and BM sterols [37], can lead to a substantial decrease in the activity levels of serum ALT and AST. In this study, following a high-starch diet, we observed increase in ALT and AST levels both in carp serum; however, these changes did not achieve statistical significance. Notably, in the group receiving 1% BMP, there was a statistically significant decrease in serum AST activity compared to the high-starch group (Figure 1). This finding aligns with previous research, suggesting that BMP alleviates liver damage in carp induced by high sugar diet to some extent.

T-AOC and T-SOD are critical indicators of the body's antioxidant capacity. Studies in mammals have shown that after 4 weeks of continuous gavage with 150 μg/g of BM polysaccharide, MDA content in mouse livers significantly decreased [38]. Similarly, after 6 weeks of gavage with 100 μg/g of BM polysaccharide, MDA content in rat serum and liver significantly decreased, while antioxidant capacity was enhanced [39]. Research in fish has also shown that under high-carbohydrate feeding, T-SOD content in the gills of largemouth bass significantly decreased, and MDA content significantly increased, indicating reduced antioxidant capacity in the fish [40]. Furthermore, after feeding common carp with high-starch feed, MDA content in their intestines significantly increased; however, upon adding 0.16% BM saponins, MDA content significantly decreased, with no significant changes in T-AOC or T-SOD levels [33]. Similarly, in this study, serum/hepatopancreas MDA content in carp was elevated following a high-starch feed. However, the addition of an appropriate amount of BMP reversed the concentrations of these two indicators (Figure 2). These results suggest that BMP may mitigate lipid peroxidation, thus, contributing to the antioxidant process in these fish.

### 4.3. BMP Can Improve the Low Polyunsaturated FAs (PUFAs) Levels in Carp Muscle Induced by a High-Starch Diet

Moisture, crude fat, crude protein, and ash are common nutritional components that reflect muscle quality. Previous research has demonstrated that the incorporation of BM seeds into the diets of grass carp results in a notable reduction in muscle fat content, accompanied by a significant increase in moisture content [41]. In this study, regarding the whole fish, no notable alterations were observed in the content of crude fat and moisture following high-starch feeding. However, the incorporation of 0.5% BMP resulted in a notable reduction in moisture content, accompanied by a substantial increase in crude fat content. Unlike the nutritional composition of the whole fish, muscle tissue exhibited an increase in ash content due to high-starch feeding, but this value returned to the control level after BMP supplementation (Table 4). These results differ from those observed in grass carp, which may be attributed to variations in the specific BM organs used in different experiments. The active components in BM seeds and flesh may also differ, and factors such as fish species and size could further influence the experimental outcomes.

Unlike livestock and poultry, fish muscle is rich in PUFAs, enhancing its unique nutritional value. Research shows that BM contains about 64.3% PUFAs [42]. Studies in mammals have indicated that incorporating dried BM fruit into the diet can reduce rats' liver n-3 PUFAs content while increasing n-6 PUFAs content [43]. In this study, high-starch feeding significantly reduced C20:3*n*−6, C20:3*n*−3, C22:6*n*−3 (DHA), and total *ω*−3 PUFAs levels in carp muscle. However, BMP supplementation increased these FAs, with the most pronounced effects on C20:3*n*−6 and C22:6*n*−3 (Table 5). These findings indicate that BMP enhances carp muscle quality by elevating these key PUFAs.

The hepatopancreas is the primary site of FA metabolism. In this study, 0.5% BMP supplementation significantly increased the concentration of hepatopancreas C20:4*n*−6, C22:6*n*−3, ∑PUFA, and ∑*ω*−3 PUFAs levels compared to those in the control cohort, while the ∑PUFA/∑SFA ratio returned to normal. This suggests that BMP promotes FA metabolism, helping maintain lipid homeostasis in the hepatopancreas. The intestine plays a key role in lipid digestion. Under high-starch conditions, intestinal levels of C20:2, C20:3*n*−3, C20:3*n*−6, C22:6*n*−3, and ∑*ω*−3 PUFAs significantly decreased. However, BMP supplementation restored or increased these FAs to control levels, indicating its role in regulating intestinal unsaturated FA metabolism (Supporting Information: Tables S1 and S2). Thus, we hypothesize that BMP may enhance DHA accumulation in both the hepatopancreas and intestine, facilitating its transport to muscle tissue.

### 4.4. BMP Reduces Glycogen and Lipid Accumulation in Carp, While Alleviating Hepatopancreatic and Intestinal Damage Caused by a High-Starch Diet

Glycogen is a vital energy reserve synthesized when energy supply is sufficient. Under high-sugar diets, liver glycogen levels significantly increase in blunt snout bream and largemouth bass [44]. BM has been reported to inhibit glycogen and lipid synthesis [45, 46]. In present study, after 8 weeks of high-starch feeding, liver and muscle glycogen levels in carp significantly increased, indicating the conversion of excess carbohydrates into glycogen. However, BMP supplementation restored glycogen levels to control levels (Figure 3), suggesting its potential to mitigate glycogen accumulation in carp, consistent with previous findings.

As dietary carbohydrate levels increase, largemouth bass exhibit lipid accumulation [1]. Similarly, HE staining of carp hepatopancreas sections in this study showed pronounced lipid accumulation under high-starch feeding, marked by severe hepatocyte vacuolation and nuclear displacement. However, BMP supplementation progressively restored hepatocyte morphology (Figure 4), suggesting its potential role in regulating lipid accumulation in the carp hepatopancreas.

The length and width of intestinal villi determine the contact area between the intestine and nutrients. Along with the thickness of the intestinal muscularis, they influence the intestinal barrier and, to some extent, reflect both the digestive capacity of the intestine and the overall health of the fish's gut [47]. Earlier research has demonstrated that high-carbohydrate feed significantly reduces intestinal villus length both in common carp [48] and tilapia [49]. In this research, as opposed to the control cohort, high-starch feeding significantly reduced the intestinal villus length in carp and increased the thickness of the muscularis, indicating that a high-starch diet may impair the digestive capacity of the carp intestine. However, after the addition of an appropriate amount of BMP, the morphology of the intestinal villi in carp was restored to control levels (Figure 4), suggesting that BMP supplementation in high-starch diets may help improve the intestinal structure and protect intestinal health.

### 4.5. BMP Regulates GLU and Lipid Metabolism in Carp by Upregulating Genes Associated With Glycolysis, While Inhibiting Those Involved in Glycogen Synthesis, Lipid Synthesis, and Transport

Previous studies have demonstrated that high-carbohydrate diets significantly upregulate glycolysis-related gene expression in the hepatopancreas of blunt snout bream [50, 51]. Similarly, in common carp, high-sugar feeding increases the expression of *pepck* and *glut2* in the hepatopancreas [52]. Additionally, supplementing high-carbohydrate feed with 1600 mg/kg of BM saponins significantly upregulates the expression of glycolysis genes (*hk*, *pk*, and *pfk*), while downregulating the expression of gluconeogenesis genes (*g6pase* and *pepck*). Consistent with these findings, this study demonstrates that high-starch feeding promotes the expression of *gys*, *pepck*, and *g6pase*. However, BMP supplementation significantly reduces the expression of glycogen synthesis-related enzymes, corresponding with observed decreases in hepatic and muscle glycogen content. Meanwhile, high-starch feeding significantly inhibited the expression of glycolysis-related genes (*gk*, *pk*, and *pygl*) in hepatopancreas, muscle, and abdominal fat, whereas BMP supplementation restores their expression to varying degrees (Figure 5). In summary, BMP may help lower blood GLU levels in carp by inhibiting glycogen synthesis-related gene expression while promoting the expression of genes involved in glycogen breakdown.

Following high carbohydrate intake, excess GLU is initially stored as glycogen in the liver and muscles. However, due to the limited glycogen storage capacity, surplus is converted into lipids, leading to fat accumulation [53, 54]. Research in fish has shown that high-carbohydrate feeding significantly upregulates lipid synthesis genes in the hepatocytes of largemouth bass [1]. In mammals, BM and its extract downregulate *srebp-1* expression in rat liver [55] and enhance intracellular FABP expression in mouse liver [56]. Similarly, the findings of this study in carp indicate that a diet high in starch markedly upregulated the mRNA abundance of critical lipid metabolism genes, including those involved in lipid synthesis (*fasn*, *acacβ*, *srebp-2*, and *pparγ*), lipid breakdown (*atgl* and *pparα*), and FA transport (*cd36*) in the hepatopancreas, intestine, and fat. BMP supplementation reduced or restored these expression levels to control values, suggesting that BMP not only suppresses lipid synthesis but also regulates lipid breakdown to maintain lipid homeostasis. In muscle tissue, high-starch feeding significantly decreased *atgl* expression, while *hsl* remained unchanged. However, BMP supplementation (0.5% and 1%) significantly upregulated both genes (Figures 6 and 7), indicating its role in promoting lipid breakdown and mitigating lipid accumulation. BMP supplementation also significantly increased the transcriptional activity of hepatopancreas *fabp1b*, *fabp10a*, and *fabp10b* (Figure 7), suggesting its role in enhancing FABP-mediated binding of free FAs, thereby contributing to GLU metabolism regulation.

After a high carbohydrate load, the maintenance of GLU and lipid metabolism homeostasis involves multiorgan coordination and the intricate interaction of complex molecular networks [57, 58]. Integrated gene correlation analysis in the hepatopancreas revealed that the expression levels of key lipid synthesis regulatory factors (*fasn*, *acacaβ*, *fads2a*, and *fads2b*) were negatively correlated with the glycolytic rate-limiting enzyme gene (*gk*), but positively correlated with key regulatory genes of gluconeogenesis (Figure 8). These findings suggest that, under carbohydrate loading, common carp may compensatorily upregulate lipid synthesis and gluconeogenesis while inhibiting glycolysis, thus, forming a dynamic mechanism for metabolic homeostasis regulation. Nonetheless, the precise molecular mechanisms that govern this process are not yet fully understood and require additional systematic investigation.

## 5. Conclusion

This study examined the impact of BMP as a feed additive on the growth characters, muscle nutrition, GLU, and lipid metabolism as well as associated gene expression in carp under high-starch feeding conditions. The results indicated that BMP had minimal impact on carp growth performance. However, it significantly reduced blood GLU and lipid levels, alleviated glycogen deposition and lipid accumulation caused by high sugar intake, and mitigated hepatopancreas and intestinal damage. BMP also increased the content of C20:3*n*−6 and C22:6*n*−3 in carp muscle. BMP reduced hepatic and muscle glycogen deposition by upregulating glycogen breakdown genes and downregulating those involved in glycogen synthesis. It also maintained the equilibrium of lipid metabolism by suppressing the transcription of genes associated with lipid synthesis and transport that are elevated in response to high-starch diets. The results of this study offer initial support for the hypothesis that BMP may enhance intestinal health while simultaneously decreasing glycogen and lipid excess deposition in both the hepatopancreas and muscle tissues. The study also suggests potential regulatory mechanisms through which BMP affects GLU and lipid metabolism in carp. However, the active components of BMP and their molecular mechanisms remain unclear. Future research should focus on identifying BMP's active components and exploring their interaction with relevant signaling and metabolic pathways to clarify their mechanisms of action.

## Figures and Tables

**Table 1:** Feed formulation and nutritional composition.

Component	Experimental group				
	C	HG	HG + 0.5% BMP	HG + 1% BMP	HG + 1.5% BMP
Ingredients (g/kg)					
Soybean meal	80	80	80	80	80
Fish meal	185	185	185	185	185
Poultry meal	80	80	80	80	80
Corn gluten meal	165	165	165	165	165
Maize starch	150	302	302	302	302
Wheat flour	110	110	110	110	110
Soybean oil	32	32	32	32	32
Ca(H_2_PO_4_)_2_	20	20	20	20	20
Microcrystalline cellulose	167	15	10	5	0
Antioxidant	1	1	1	1	1
Premix compound	10	10	10	10	10
Carboxy methyl cellulose	15	15	15	15	15
Bitter melon powder	0	0	5	10	15
Nutrient levels (%)					
Crude protein	33.83	32.96	32.72	33.38	32.29
Moisture	8.47	8.80	8.92	9.11	8.93
Crude lipid	6.06	6.26	6.37	6.44	6.36
Ash	7.72	8.07	7.52	7.91	7.18
Crude fiber	16.09	3.52	3.16	3.14	3.68
Nitrogen-free extract	27.83	40.39	41.31	40.02	41.56

**Table 2:** Primers for RT-qPCR assays.

Gene	Forward	Reverse	Entry number
*gys*	TTTTGGCCGCTGGTTGATTG	ATAGGGTAGTCCAATGCTGCAC	XM_019090903.1
*pepck*	ATGGCGTGTTTGTAGGAGCAG	GGGCCAAGTAGTCACCGAAGT	KP250869
*g6pase*	CTGGGTGGCTGTGATAGGAGAC	ATGTGGCGTTGAGCTGTTGAT	XM_019101462
*glut2*	CAGATGAGTGTGAGTCCGCAGAAG	CCATAGGCTGGTCCTCAGAGTCA	XM_019064582.1
*gk*	ACTGTGACATTGTGCGTCTGGT	CTTGACAACGGCGTTCCCT	AF053332
*pk*	GATATGGGCTCTGCCTTCAT	TCCGATGGTGCAGATGATTC	XM_019112746
*pfk*	CGTTCGACAGGAATTTTGGC	TTCATGCCGATCACACAAGC	XM_042766957.1
*pygl*	TGGTTGACGACGATGCTTTC	ACTGCGCAAACTTCAGCTTG	XM_019125106.1
*fasn*	TCTGTGCTGTGCGGACTGGAA	GCAACATCGGCTGGATATTGAGGAG	KY378913.1
*acacβ*	ACTGGCTGGCTAGATCACCTTATTG	GCACCGCATACCACACCTAACAT	XM_019106587.1
*srebp-1*	CACGGCTCTGCTCAACGACAT	TGCGGAGGAGACTGCTGGAA	XM_019073316.1
*srebp-2*	CAGATGAGTGTGAGTCCGCAGAAG	CCATAGGCTGGTCCTCAGAGTCA	XM_019064582.1
*pparγ*	GCAAGGCAGTGGAGGACAAGAAC	ACGCAACACAGCACCATAAGAGG	LOC109050306
*fads2a*	CTGTAACATTGAGCAATCCGCCTTC	GGTACGCATCCAGCCAGAGTTC	MK852165.1
*fads2b*	AAGTAAGCAGCAGTCAGTCAGAGTT	CCGTGGCATCTTCTCCAGCATAG	MK852166.1
*atgl*	CACCAACACCTCCATTCAGTTCACA	ACTCTTCATCCTCCTCACCGTCAG	KY906167.1
*hsl*	TTGATGCCTATGCTGGTACGAGTTG	TGATGTGGTTGGAGAGGATGATGCT	MF061228.2
*pparα*	GCATGAAGCCTACCTCAGACACTT	ACCGAGGCGTACTGGCAGAA	FJ849065.1
*cd36*	CCGTAGGCACAGAGGAGGACATAT	GAGTGTGGAATTGGAGCGTTGGA	KM030422.1
*fabp1b*	ATCGAATCCCTGACCGGAGA	CCTCTTGTAGACGAGGCTGC	XM_019124255.1
*fabp10a*	AACCCCTGGAAAAACCGTCA	GGGTCTCCACCATCTCTCCT	EU363800.1
*fabp10b*	AACTCCTTCACCATCGGCAA	CTCCTACCGTCAGGGTCTCC	EU363801.1
*β-actin*	TGCAAAGCCGGATTCGCTGG	AGTTGGTGACAATACCGTGC	FJ710827.1

**Table 3:** Effects of BMP on the growth and morphological indices.

Index	C	HG	HG + 0.5% BMP	HG + 1% BMP	HG + 1.5% BMP
Initial weigh (g)	3.06 ± 0.02	3.06 ± 0.02	3.06 ± 0.02	3.06 ± 0.02	3.06 ± 0.02
Final weigh (g)	11.11 ± 0.72	10.46 ± 0.66	11.43 ± 0.32	11.03 ± 0.54	10.12 ± 0.05
WGR (%)	264.16 ± 23.72	242.49 ± 21.65	273.94 ± 10.35	260.82 ± 17.47	231.10 ± 1.59
SGR (%)	2.30 ± 0.12	2.19 ± 0.12	2.35 ± 0.05	2.29 ± 0.08	2.14 ± 0.01
FCR	1.67 ± 0.15	1.83 ± 0.17	1.60 ± 0.06	1.69 ± 0.11	1.87 ± 0.01
SR (%)	100 ± 0.00	97.78 ± 1.11	95.56 ± 1.11	98.89 ± 1.11	98.89 ± 1.11
VSI (%)	6.55 ± 0.18^a^	6.42 ± 0.19^a^	6.41 ± 0.17^a^	6.25 ± 0.11^a^	5.64 ± 0.14^b^
HSI (%)	1.98 ± 0.10^bc^	2.45 ± 0.10^a^	2.06 ± 0.08^bc^	2.22 ± 0.08^ab^	1.90 ± 0.07^c^
CF (g/cm^3^)	2.56 ± 0.03^b^	2.65 ± 0.03^ab^	2.65 ± 0.04^ab^	2.68 ± 0.03^a^	2.72 ± 0.03^a^

*Note:* The data are expressed as the mean ± SEM, with a sample size of *n* = 9 for each tank. Values that are denoted with different superscript letters signify statistically significant differences (*p* < 0.05). CF = Final weight (g)/final body length^3^ (cm^3^) × 100; FCR = feed intake (g)/(final weight − initial weight) (g); HSI = wet hepatopancreas weight (g)/final fish weight (g) × 100; SGR = [Ln (final weight) (g) − Ln (initial weight) (g)]/days of feeding × 100; SR = survival quantity (g)/initial quantity (g) × 100; VSI = wet viscera weight (g)/wet fish weight (g) × 100; WGR = (final weight − initial weight) (g)/initial weight (g) × 100.

Abbreviations: CF, coefficient of fatness; FCR, feed conversion ratio; HSI, hepatosomatic index; SGR, specific growth rate; SR, survival rate; VSI, visceral index; WGR, weight gain rate.

**Table 4:** Effects of BMP on whole fish and muscle routine components in common carp.

Contents	Experimental groups				
	C	HG	HG + 0.5% BMP	HG + 1% BMP	HG + 1.5% BMP
Whole body composition (%)					
Moisture	75.53 ± 0.40^ab^	75.66 ± 0.15^a^	74.50 ± 0.19^c^	74.71 ± 0.25^bc^	74.79 ± 0.39^abc^
Crude lipid	6.06 ± 0.25^c^	6.48 ± 0.13^bc^	7.17 ± 0.07^a^	6.94 ± 0.15^ab^	7.01 ± 0.23^ab^
Crude protein	13.54 ± 0.34	12.80 ± 0.30	13.43 ± 0.29	13.28 ± 0.16	13.18 ± 0.25
Ash	8.78 ± 0.85	8.49 ± 0.18	8.96 ± 0.69	8.43 ± 0.36	7.97 ± 0.54
Muscle composition (%)					
Moisture	70.88 ± 2.79	72.00 ± 0.16	72.60 ± 0.40	72.77 ± 0.75	71.51 ± 0.38
Crude lipid	0.64 ± 0.06	0.71 ± 0.03	0.69 ± 0.05	0.66 ± 0.04	0.70 ± 0.02
Crude protein	25.24 ± 0.30	23.71 ± 0.29	24.80 ± 1.17	23.68 ± 0.25	24.44 ± 0.28
Ash	6.81 ± 0.43^b^	8.20 ± 0.51^a^	7.91 ± 0.43^ab^	7.26 ± 0.34^ab^	7.44 ± 0.03^ab^

*Note:* Sample size is ≥3 for each group. The data and significant differences are consistent with those presented in Table 3.

**Table 5:** Effects of BMP on muscle fatty acid profile.

FAs	Experimental groups				
	C	HG	HG + 0.5% BMP	HG + 1% BMP	HG + 1.5% BMP
C14:0	0.67 ± 0.02	0.68 ± 0.01	0.65 ± 0.01	0.64 ± 0.01	0.67 ± 0.02
C16:0	22.85 ± 0.29^b^	22.48 ± 0.23^b^	22.49 ± 0.21^b^	22.70 ± 0.19^b^	23.66 ± 0.03^a^
C16:1	3.64 ± 0.18^ab^	3.93 ± 0.05^a^	3.76 ± 0.10^ab^	3.59 ± 0.01^b^	3.73 ± 0.07^ab^
C18:0	10.11 ± 0.43^b^	9.67 ± 0.12^b^	9.66 ± 0.30^b^	10.23 ± 0.14^b^	11.33 ± 0.23^a^
C18:1	27.62 ± 0.54^b^	31.36 ± 0.31^a^	30.74 ± 0.35^a^	30.83 ± 0.70^a^	28.39 ± 0.34^b^
C18:2*n*−6	20.22 ± 0.52^a^	18.14 ± 0.22^b^	18.22 ± 0.20^b^	17.64 ± 0.07^b^	18.18 ± 0.21^b^
C18:3*n*−6	0.41 ± 0.02^ab^	0.42 ± 0.01^ab^	0.43 ± 0.01^a^	0.39 ± 0.02^b^	0.41 ± 0.01^ab^
C18:3*n*−3	2.34 ± 0.10^a^	2.08 ± 0.02^b^	2.07 ± 0.04^b^	2.01 ± 0.02^b^	1.98 ± 0.02^b^
C20:0	1.64 ± 0.05	1.71 ± 0.03	1.68 ± 0.01	1.72 ± 0.02	1.62 ± 0.03
C20:2	0.95 ± 0.07	0.93 ± 0.01	0.96 ± 0.02	0.97 ± 0.01	0.93 ± 0.01
C20:3*n*−6	1.67 ± 0.08^a^	1.51 ± 0.01^b^	1.56 ± 0.03^ab^	1.61 ± 0.04^ab^	1.67 ± 0.01^a^
C20:4*n*−6	3.29 ± 0.16	3.24 ± 0.03	3.55 ± 0.13	3.51 ± 0.16	3.53 ± 0.05
C20:3*n*−3	0.12 ± 0.01^a^	0.08 ± 0.01^b^	0.09 ± 0.01^b^	0.09 ± 0.01^ab^	0.09 ± 0.01^ab^
C20:5*n*−3	0.54 ± 0.03^a^	0.44 ± 0.04^b^	0.44 ± 0.02^b^	0.31 ± 0.02^c^	0.29 ± 0.01^c^
C22:6*n*−3	3.92 ± 0.16^a^	3.33 ± 0.07^b^	3.72 ± 0.15^ab^	3.75 ± 0.18^a^	3.52 ± 0.05^ab^
∑SFA	35.27 ± 0.71^b^	34.54 ± 0.37^b^	34.47 ± 0.49^b^	35.29 ± 0.29^b^	37.28 ± 0.21^a^
∑PUFA	30.16 ± 0.37^a^	26.93 ± 0.21^b^	27.48 ± 0.13^b^	26.77 ± 0.30^b^	27.07 ± 0.19^b^
∑PUFA/∑SFA	0.86 ± 0.03^a^	0.78 ± 0.01^b^	0.80 ± 0.01^b^	0.76 ± 0.01^bc^	0.73 ± 0.01^c^
∑*ω*−6	22.41 ± 0.44^a^	20.15 ± 0.21^b^	20.29 ± 0.18^b^	19.73 ± 0.12^b^	20.35 ± 0.22^b^
∑*ω*−3	6.80 ± 0.06^a^	5.85 ± 0.04^c^	6.23 ± 0.13^b^	6.07 ± 0.18^bc^	5.79 ± 0.04^c^
∑*ω*−6/∑*ω*−3	3.30 ± 0.09^ab^	3.45 ± 0.04^ab^	3.26 ± 0.09^b^	3.26 ± 0.08^b^	3.51 ± 0.05^a^

*Note:* Sample size is 9 for each group. The data and significant differences are consistent with those presented in Table 3. SA represents the total content of C14:0, C16:0, C18:0, and C20:0; PUFA refers to the total of C18:2*n*−6, C18:3*n*−6, C18:3*n*−3, C20:2, C20:3*n*−6, C20:4*n*−6, C20:5*n*−3, and C22:6*n*−3; ∑*ω*−3 is the sum of C18:3*n*−3, C20:5*n*−3, and C22:6*n*−3; ∑*ω*−6 is the sum of C18:2*n*−6, C18:3*n*−6, C20:3*n*−6, and C20:4*n*−6.

## Data Availability

The data will be made available upon request.

## Supporting Information

Supporting Information Table S1. Effects of BMP on hepatopancreas fatty acid profile. Table S2. Effects of BMP on intestinal fatty acid profile.
