# Supplementary material for: Bitter Melon Powder Enhances Antioxidant Capacity, Muscle Nutrition, and Glucolipid Metabolic Homeostasis in Cyprinus carpio Fed High-Starch Diets
Source: Aquac Nutr. 2025 Nov 29;2025:9209833. doi: 10.1155/anu/9209833 (PMC12681423; doi:10.1155/anu/9209833)
Supplement: Supporting Information — Table S1. Effects of BMP on hepatopancreas fatty acid profile. Table S2. Effects of BMP on intestinal fatty acid profile. [file 9209833.f1.docx]

**Table S1 Effects of BMP on hepatopancreas fatty acid profile**

| **FAs** | **experimental groups** | | | | |
| --- | --- | --- | --- | --- | --- |
|  | **C** | **HG** | **HG+**  **0.5%BMP** | **HG+**  **1%BMP** | **HG+**  **1.5%BMP** |
| C14:0 | 1.00±0.07^a^ | 1.08±0.07^a^ | 0.86±0.01^b^ | 0.98±0.01^ab^ | 1.02±0.03^a^ |
| C16:0 | 25.89±0.44^a^ | 23.47±0.17^b^ | 23.89±0.72^b^ | 23.50±0.40^b^ | 22.60±0.12^b^ |
| C16:1 | 5.74±0.39 | 6.05±0.52 | 5.20±0.07 | 5.52±0.14 | 5.63±0.20 |
| C18:0 | 12.49±0.89^a^ | 9.63±0.64b^c^ | 11.28±0.61^ab^ | 10.49±0.59^abc^ | 8.98±0.76^c^ |
| C18:1 | 36.35±1.58^b^ | 40.14±1.35^ab^ | 36.49±1.82^b^ | 43.03±1.05^a^ | 41.96±1.60^a^ |
| C18:2n6 | 9.82±0.48 | 10.70±1.00 | 11.20±0.64 | 9.62±0.50 | 10.72±0.14 |
| C18:3n6 | 0.20±0.01^bc^ | 0.22±0.01^abc^ | 0.24±0.02^a^ | 0.18±0.02^c^ | 0.23±0.01^ab^ |
| C18:3n3 | 0.77±0.04^b^ | 0.94±0.10^ab^ | 1.06±0.09^a^ | 0.08±0.04^b^ | 0.95±0.02^ab^ |
| C20:0 | 2.38±0.13^ab^ | 2.37±0.09^ab^ | 2.12±0.08^b^ | 2.18±0.03^ab^ | 2.44±0.06^a^ |
| C20:2 | 0.65±0.01^a^ | 0.60±0.02^a^ | 0.68±0.03^a^ | 0.46±0.03^b^ | 0.68±0.06^a^ |
| C20:3n6 | 0.76±0.07 | 0.79±0.09 | 0.83±0.05 | 0.67±0.05 | 0.68±0.09 |
| C20:4n6 | 1.61±0.22^b^ | 1.78±0.15^b^ | 2.62±0.33^a^ | 1.20±0.06^b^ | 1.85±0.29^b^ |
| C20:3n3 | 0.03±0.006^a^ | 0.02±0.003^a^ | 0.02±0.002^ab^ | 0.01±0.001^b^ | 0.03±0.005^a^ |
| C20:5n3 | 0.37±0.06^a^ | 0.26±0.02^ab^ | 0.33±0.06^ab^ | 0.29±0.02^ab^ | 0.22±0.02^b^ |
| C22:6n3 | 1.78±0.27^b^ | 1.94±0.17^b^ | 3.18±0.56^a^ | 1.05±0.10^b^ | 2.02±0.39^b^ |
| ∑SA | 41.76±1.13^a^ | 36.55±0.70^b^ | 38.15±1.25^b^ | 37.16±0.95^b^ | 35.04±0.81^b^ |
| ∑PUFA | 14.54±0.72^b^ | 15.48±1.28^ab^ | 17.54±0.68^a^ | 13.09±0.59^b^ | 15.51±0.67^ab^ |
| ∑PUFA/∑SA | 0.35±0.01^b^ | 0.42±0.03^a^ | 0.46±0.02^a^ | 0.35±0.02^b^ | 0.44±0.01^a^ |
| ∑ω-6 | 10.96±0.56 | 11.73±1.05 | 12.29±0.63 | 10.49±0.46 | 11.65±0.23 |
| ∑ω-3 | 2.92±0.28^b^ | 3.14±0.24^b^ | 4.57±0.57^a^ | 2.14±0.14^b^ | 3.19±0.39^b^ |
| ∑ω-6/∑ω-3 | 3.87±0.33^b^ | 3.73±0.15^b^ | 2.92±0.38^b^ | 4.95±0.24^a^ | 3.90±0.40^b^ |

Data are presented as the mean±SEM (n =9 for each group). Values with different superscript letters indicate significant differences (P < 0.05). SA = C14:0 + C16:0 + C18:0 + C20:0; PUFA = C18:2n6 + C18:3n6 + C18:3n3 + C20:2 + C20:3n6 + C20:4n6 + C20:5n3 + C22:6; ∑ω-3 = C18:3n3 + C20:5n3 + C22:6n3; ∑ω-6 = C18:2n6 + C18:3n6 + C20:3n6 + C20:4n6.

**Table S2 Effects of BMP on fatty acid composition in intestinal of common carp**

| **contents** | **experimental groups** | | | | |
| --- | --- | --- | --- | --- | --- |
|  | **C** | **HG** | **HG+0.5%BMP** | **HG+1%BMP** | **HG+1.5%BMP** |
| C14:0 | 0.81±0.05^b^ | 0.96±0.02^a^ | 0.92±0.01^a^ | 0.89±0.04^ab^ | 0.90±0.07^ab^ |
| C16:0 | 24.43±0.92^ab^ | 23.42±0.71^abc^ | 25.08±0.61^a^ | 22.42±0.10^c^ | 22.86±0.16^bc^ |
| C16:1 | 3.42±0.29^b^ | 3.94±0.04^a^ | 3.56±0.11^ab^ | 3.77±0.09^ab^ | 3.75±0.07^ab^ |
| C18:0 | 12.62±1.22^a^ | 9.54±0.38^b^ | 10.11±1.24^ab^ | 9.48±0.44^b^ | 10.22±0.21^ab^ |
| C18:1 | 26.83±1.53^b^ | 32.80±0.26^a^ | 32.75±0.72^a^ | 31.07±0.11^a^ | 30.50±0.63^a^ |
| C18:2n6 | 20.49±1.16^a^ | 19.73±0.79^ab^ | 17.88±0.59^b^ | 21.22±0.82^a^ | 20.78±0.51^a^ |
| C18:3n6 | 0.32±0.01 | 0.30±0.04 | 0.27±0.01 | 0.30±0.02 | 0.25±0.04 |
| C18:3n3 | 2.12±0.19^ab^ | 2.13±0.19^ab^ | 1.73±0.11^b^ | 2.30±0.14^a^ | 2.17±0.07^ab^ |
| C20:0 | 1.28±0.03^c^ | 1.53±0.03^ab^ | 1.61±0.04^a^ | 1.43±0.04^b^ | 1.43±0.04^b^ |
| C20:2 | 0.71±0.04^a^ | 0.66±0.02^b^ | 0.68±0.01^ab^ | 0.75±0.02^a^ | 0.75±0.02^a^ |
| C20:3n6 | 1.11±0.13^a^ | 0.81±0.07^b^ | 0.83±0.02^b^ | 1.00±0.06^ab^ | 1.00±0.06^ab^ |
| C20:4n6 | 2.50±0.40 | 1.92±0.14 | 2.00±0.05 | 2.45±0.17 | 2.45±0.15 |
| C20:3n3 | 0.07±0.006^a^ | 0.05±0.004^bc^ | 0.05±0.003^c^ | 0.06±0.001^ab^ | 0.06±0.002^bc^ |
| C20:5n3 | 0.42±0.04^a^ | 0.35±0.03^ab^ | 0.30±0.02^b^ | 0.31±0.02^b^ | 0.33±0.03^b^ |
| C22:6n3 | 2.83±0.52^a^ | 1.88±0.12^b^ | 2.23±0.06^ab^ | 2.55±0.19^ab^ | 2.55±0.21^ab^ |
| ∑SA | 39.14±2.05^a^ | 35.45±1.13^ab^ | 37.73±1.39^ab^ | 34.22±0.49^b^ | 35.41±0.36^ab^ |
| ∑PUFA | 28.12±0.64^ab^ | 25.90±1.20^bc^ | 23.97±0.67^c^ | 28.49±0.67^a^ | 27.88±0.71^ab^ |
| ∑PUFA/∑SA | 0.73±0.06^ab^ | 0.74±0.06^ab^ | 0.64±0.04^b^ | 0.83±0.03^a^ | 0.79±0.03^a^ |
| ∑ω-6 | 21.1±1.02^a^ | 20.89±0.90^ab^ | 19.03±0.61^b^ | 22.59±0.75^a^ | 22.08±0.57^a^ |
| ∑ω-3 | 5.38±0.36^a^ | 4.36±0.30^bc^ | 4.26±0.07^c^ | 5.17±0.06^a^ | 5.06±0.28^ab^ |
| ∑ω-6/∑ω-3 | 4.26±0.52 | 4.84±0.16 | 4.46±0.10 | 4.39±0.20 | 4.42±0.23 |

Data are presented as the mean±SEM (n =9 for each group). Values with different superscript letters indicate significant differences (P < 0.05) (n = 3). SA = C14:0 + C16:0 + C18:0 + C20:0; PUFA = C18:2n6 + C18:3n6 + C18:3n3 + C20:2 + C20:3n6 + C20:4n6 + C20:5n3 + C22:6; ∑ω-3 = C18:3n3 + C20:5n3 + C22:6n3; ∑ω-6 = C18:2n6 + C18:3n6 + C20:3n6 + C20:4n6.
